# Antenatal Maternal Smoking and Lung Function in Very Prematurely Born Children

**DOI:** 10.1002/ppul.71572

**Published:** 2026-03-15

**Authors:** Allan Jenkinson, Sanja Zivanovic, Christopher Harris, Theodore Dassios, Anne Greenough

**Affiliations:** ^1^ Department of Neonatology, Neonatal Intensive Care Unit King's College Hospital NHS Foundation Trust London UK; ^2^ Department of Women and Children's Health School of Life Course and Population Sciences, Faculty of Life Sciences and Medicine, King's College London London UK; ^3^ Department of Neonatology Imperial College Healthcare NHS Trust London UK

## Abstract

**Introduction:**

Antenatal exposure to maternal smoking negatively impacts on fetal lung development resulting in infant lung function abnormalities. Lung clearance indices (LCI) were worse in term born infants exposed to antenatal maternal smoking compared to not. Our aim was to assess the effect of antenatal maternal smoking on lung function, in particular LCI, in very prematurely born children.

**Methods:**

Lung function testing was undertaken at 11–14 years, when household members smoking and active smoking were assessed. Antenatal maternal smoking had been recorded. Lung function was assessed by LCI, spirometry, plethysmography, gas transfer and fraction of exhaled nitric oxide.

**Results:**

Two hundred and thirty children with a median gestation age of 27 weeks were assessed. Fifty‐five had antenatal exposure to maternal smoking. Those with exposure to antenatal maternal smoking were more likely to live with a household member that smoked (67% vs. 18%, *p* < 0.001). Children of mothers who smoked in pregnancy were also more likely to be active smokers at follow up (indicated by urinary cotinine levels greater than 15 ng/mL) (49% vs. 7%; *p* < 0.001). Lung clearance index was higher in children exposed to maternal smoking in pregnancy (mean (SD): 7.8 (1.1) vs. 7.4 (1.2); mean difference (95% CI): −0.44 (−0.91, 0.02); *p* = 0.031). There were no significant differences in other measures of lung function.

**Conclusion:**

Very prematurely born children exposed to antenatal maternal smoking had greater ventilation inhomogeneity and were more likely to be active smokers and live with a household member that smoked. These findings were not explained by active smoking or household smoking contact at follow up, but those who had BPD had greater ventilation inhomogeneity.

## 1 Introduction

Maternal smoking during pregnancy is associated with adverse health outcomes in their offspring. Exposure to antenatal maternal smoking is associated with impaired fetal growth, pre‐term birth and lower birth weight [1]. Furthermore, children exposed to maternal smoking during pregnancy were more likely to have an increased respiratory morbidity, with a higher incidence of bronchiolitis, asthma and hospital admissions for respiratory infections [2]. Exposed infants also have reduced lung function, as evidenced by reduced static compliance in boys and conductance in girls born at term and less than 72 h of age [3]. Other lung function abnormalities include a reduced functional residual capacity at 1 year [4] and reduced forced expiratory flow in adolescence [5]. Furthermore, lung clearance indices (LCI) were worse in term born infants exposed to antenatal maternal smoking compared to unexposed term infants [6].

The lung clearance index (LCI) is an index of ventilation inhomogeneity in the peripheral airways [7]. Inhomogeneity indices may be more sensitive than spirometry or other lung function assessments for the early detection of small airway disease [8]. Children with cystic fibrosis aged between 2 and 5 years had significantly higher lung clearance index compared to healthy controls (mean [95% CI] difference 2.7 [1.9, 3.6], *p* < 0.001). In that cohort, lung function abnormalities were detected in 73% of the cohort by LCI, 47% by plethysmography and 13% by spirometry.

The aim of this study was to determine whether antenatal maternal smoking exposure was associated with abnormal lung function including lung clearance index in extremely prematurely born children when assessed at 11–14 years.

## 2 Methods

Young people who had been recruited into the United Kingdom Oscillation Study (UKOS) were invited to King's College Hospital NHS Foundation Trust (KCH) to undergo lung function testing when they were aged between 11 and 14 years of age [9]. Parents gave written consent for their child(ren) to participate in the study and their parents had previously given written consent for their infant to participate in the UKOS trial. Ethical approval for UKOS was granted by the Thames Multicentre Research Ethics Committee [10] and for the follow‐up study by the North‐East–Tyne & Wear South Research Ethics Committee (16/NE/0314) [9].

Lung function testing was performed according to European Respiratory Society guidelines. A multiple breath washout technique using sulfur hexafluoride was used to assess the LCI. Spirometry, including FEV_1_, FVC, forced expired flow at 25%, 50%, 75% and between 25% and 75% of the FVC (FEF_25_, FEF_50_,FEF_75_ and FEF _25–75_), was undertaken. Lung volumes including functional residual capacity (FRC), total lung capacity (TLC) and residual volume (RV) were measured by plethysmography. A single breath washout technique was used to measure diffusion capacity of the lung for carbon monoxide. Impulse oscillometry was used to measure airways resistance at 5 and 20 Hz (R5Hz and R20Hz). Spirometric, diffusion and plethysmography lung‐function results were expressed as z‐scores [11, 12, 13]. Lung clearance index results were expressed as absolute values and FeNO expressed as parts per million (ppm) as reported in technical standards [14, 15].

Demographics (birthweight [grams], gestational age [weeks], gender [male/female], mode of ventilation [HFOV/CV], surfactant [yes/no], postnatal corticosteroid [yes/no] and BPD [yes/no]), maternal characteristics (chorioamnionitis [yes/no], antenatal corticosteroids [yes/no], maternal smoking by self report [yes/no]) were recorded in the original UKOS database [10]. At follow up, parents were asked about the presence of smokers in the house (yes/no). A urine sample for the detection of cotinine was obtained from the children at the time of assessment to assess whether they were active smokers [9]. Active smoking was indicated by a urine cotinine level > 15 ng/mL and was presented as a dichotomous variable (yes/no).

## 3 Analysis

Data were tested for normality with the Kolmogorov–Smirnov test. Data were normally distributed and therefore differences in lung function results between those with and without antenatal maternal smoking exposure were tested for statistical significance using an independent *t*‐test analysis. Correlation and regression analyses were conducted to explore factors influencing lung function including lung clearance index. As the results of those exposed to antenatal smoking were expected to be worse than those who were not, hence a one sided *t*‐test was used. Statistical analysis was performed using SPSS software, version 27.0 (SPSS Inc., Chicago, Illinois, USA).

## 4 Results

There were no significant differences in neonatal or maternal characteristics in those exposed or unexposed to maternal antenatal smoking (Table 1). Children of mothers who smoked in pregnancy were more likely to be active smokers at follow up (49% vs. 7%; *p* < 0.001) (Table 1). Additionally, children of mothers who smoked in pregnancy were more likely be living with a household member who smoked (67% vs. 18%; *p* < 0.001) (Table 1).

**Table 1:** Demographic differences.

	No maternal smoking *n* = 175	Maternal smoking *n* = 55	*p* value
Gestational Age (weeks)	27.7 (25.7–28)	27 (26.4–27.8)	0.747
Birthweight (grams)	865 (740–1050)	885 (710–1078)	0.472
Male	92 (52.6)	27 (49.1)	0.757
Antenatal corticosteroids	154 (89)	53 (96.4)	0.116
Chorioamnionitis	13 (7.4)	5 (9)	0.282
HFOV	89 (50.9)	31 (56.4)	0.537
Surfactant	168 (96)	54 (98.2)	0.684
Postnatal corticosteroids	49 (28.3)	12 (22)	0.482
BPD at 36 weeks corrected gestational age	92 (52.6)	35 (63.6)	0.164
Shared accommodation with smoker at follow up	31 (18)	37 (67)	< 0.001
Active smoking (urinary cotinine > 15 ng/mL)	12 (7)	23 (47)	< 0.001

*Note:* Data presented as median (IQR) or *n* (%).

Lung clearance index was higher in children exposed to antenatal smoking as compared to no antenatal smoking (mean (SD): 7.8 (1.1) vs. 7.4 (1.2); mean difference (95% CI): −0.44 (−0.91, 0.02); *p* = 0.031) (Table 2). There were no significant differences in spirometry, lung volume or airway resistance results between the two groups. In multivariable regression analysis, both BPD and antenatal maternal smoking had a significant association with lung clearance index; BPD had the stronger independent association (*p* = 0.019 vs. *p* = 0.088). Neither active smoking nor household smoke exposure at follow‐up were significantly associated with lung clearance index. Almost all infants were mechanically ventilated, exposed to antenatal corticosteroids and received postnatal surfactant and so not examined.

**Table 2:** Lung function results according to antenatal maternal smoking exposure.

	No maternal smoking *n* = 175	Maternal smoking *n* = 55	Mean difference (95% CI)	
FEV_1_	−0.80 (1.05)	−0.67 (1.1)	−0.13 (−0.46, 0.19)	0.214
FVC	−0.38 (0.98)	−0.28 (1.0)	−0.09 (−0.4, 0.2)	0.267
FEV_1_/FVC	−1.5 (1.80)	−1.4 (1.9)	−0.12 (−0.68, 0.43)	0.328
PEF	−0.86 (0.84)	−0.87 (0.79)	0.01 (−0.23, 0.27)	0.440
FEF_25_	−1.02 (0.96)	−0.93 (0.94)	−0.09 (−0.38, 0.20)	0.265
FEF_50_	−1.21 (0.92)	−1.21 (0.89)	−0.007 (−0.28, 0.27)	0.480
FEF_75_	−1.08 (0.88)	−1.0 (0.94)	−0.05 (−0.32, 0.22)	0.357
FEF_25–75_	−1.45 (1.0)	−1.4 (1.1)	−0.03 (−0.38, 0.30)	0.423
RV	0.46 (1.29)	0.31 (1.26)	0.15 (−0.27, 0.57)	0.241
TLC	0.31 (1.08)	0.29 (1.02)	0.01 (−0.34, 0.37)	0.457
FRC_pleth_	−0.03 (1.3)	−0.1 (1.2)	0.07 (−0.34, 0.49)	0.366
FRC_he_	−0.61 (1.1)	−0.77 (0.98)	0.15 (−0.18, 0.50)	0.187
DLCO	−0.97 (1.0)	−0.73 (1.2)	−0.24 (−0.59, 0.11)	0.090
FeNO	19.66 (18.6)	19.31 (19.5)	0.34 (−5.59, 6.5)	0.456
R5Hz	0.53 (1.04)	0.62 (0.91)	−0.08 (−0.40, 0.23)	0.299
R20Hz	0.34 (1.1)	0.35 (0.98)	−0.01 (−0.34, 0.32)	0.473
LCI	7.4 (1.2)	7.8 (1.1)	−0.44 (−0.91, 0.02)	0.031

*Note:* The lung function results are expressed as mean (SD). Results expressed as z‐score except for LCI and FeNO.

Abbreviations: CI, confidence interval; DLCO, diffusion capacity of carbon monoxide; FeNO, fraction of exhaled nitric oxide; FEF, forced expiratory flow; FEV1, forced expiratory volume in 1 s; FRC, functional residual capacity; FVC, forced vital capacity; LCI, lung clearance index; PEF, peak expiratory flow; RV, residual volume; TLC, total lung capacity.

Lung clearance index and antenatal smoking were not associated with increased morbidity, as defined by the presence or absence of wheeze, hospital admission or receiving antibiotics.

## 5 Discussion

We have demonstrated that children exposed to antenatal maternal smoking and born extremely prematurely had higher LCI when aged 11–14 years, suggesting they had greater lung inhomogeneity than those unexposed to antenatal maternal smoking. In addition, when assessed at 11–14 years they were more likely to be active smokers and have a household member who smoked, both of which may also have had an effect on their lung function.

Lum et al investigated the relationship between body size and LCI from infancy to young adulthood and established a reference range for LCI. The mean ± standard deviation LCI results for children aged 6–19 years of age was 6.54 ± 0.51, with an upper limit of normal for this age group of 7.56 [16]. We have demonstrated that children born preterm and exposed to antenatal smoking had mean LCI results above that upper limit of normal. Those results are plausible in that animal studies have shown nicotine exposure to be associated with increased collagen deposition, thickened alveolar walls and increased airway smooth muscle mass [17, 18]. In addition, the LCI results in our children not exposed to antenatal maternal smoking are consistent with previously reported results for unexposed preterm individuals in this age range [19, 20]. When examined with BPD, active smoking and exposure to second hand smoking (household contact) at follow up, only maternal smoking and BPD were associated with LCI. BPD demonstrated the strongest independent association consistent with previously published reports [21].

LCI has been used as a marker of smaller airway disease in pediatric populations with cystic fibrosis. Subararro et al reported its use in the assessment of treatment effects of inhaled isotonic and hypertonic saline in a group of preschool children with cystic fibrosis [22]. LCI measurements were performed at baseline and 48 weeks demonstrating a significant treatment effect in the hypertonic saline group (LCI z score treatment effect: 2.01; 95% CI = 0.26–3.76; *p* = 0.025). More recently, Svedberg et al correlated LCI to structural lung damage assessed on chest CT in a Swedish cohort with CF aged 0–17 years [23]. Their results demonstrated that a low LCI during childhood was associated with less structural lung damage and a slower structural lung damage progression rate compared with a higher longitudinal mean LCI [23]. Children with CF may go through childhood with only subtle upper and lower airway‐related symptoms and have well‐preserved lung function assessed by spirometry [24]. Therefore, LCI may detect early lung disease before disease progression results in abnormal spirometry. This may explain our results showing a difference in LCI between groups, but with no significant differences in other lung function results.

Antenatal smoking is more common in high as compared to low and middle income countries; it has been reported that 14%–20% of women in the UK smoked during pregnancy. Thirty‐one percent of the mothers in this study smoked during pregnancy [25]. The higher proportion may reflect that all the offspring were born extremely prematurely and antenatal smoking is known to increase premature birth [26]. Pregnancy has been suggested to be a major teachable moment to promote a healthy lifestyle given mothers' concerns about fetal health and their regular contact with healthcare providers [27]. Although there are many interventions to reduce maternal smoking and pre and postnatal second hand exposure [27], the proportion of women smoking in this study demonstrate that they have limited success or are not used.

In this study, children whose mothers smoked during pregnancy were more likely to be active smokers. Evidence of the risk of active smoking in the offspring of women who smoked during pregnancy is conflicting [28, 29, 30]. In a prospective cohort study linked to a national register, Rydell et al. found no significant association between maternal smoking and an increased risk of active smoking in offspring [28]. A sibling‐pair sub‐analysis also found no clear evidence supporting a link between maternal smoking during pregnancy and increased risk [31]. In a longitudinal cohort study in southwest England, however, maternal smoking was positively associated with offspring smoking initiation (Odd's ratio [OR] = 1.33 [95% CI = 1.06, 1.67]) [25]. Furthermore, in a 30 year prospective study, an elevated risk of tobacco dependence was found amongst offspring at a mean age 29 years of mothers who smoked at least a pack of cigarettes on 1 or more days during pregnancy [32]. Whether an intrauterine mechanism is responsible remains disputed, as partner smoking is similarly associated (OR = 1.28 [95% CI = 1.06, 1.55]) [29]. Some studies suggest that genetic and environmental factors may confound this association [31].

Adolescent smoking has been demonstrated to result in reduced FEV_1_/FVC ratios and increased peripheral airway resistance [33]. Compared to nonsmoking participants, adolescent smoking at 16 years of age was associated with significantly lower FEV_1_/FVC ratios of −0.9% (95% confidence interval [CI] −1.8% to 0.1%). Children exposed to maternal smoking who go on to smoke would therefore have a further increased risk of abnormal lung function, which may be even more pronounced in extremely preterm infants since they already have some preexisting impairment. In the present cohort, antenatal smoking exposure and lung clearance index measured at 11 years of age were not associated with increased respiratory morbidity as defined by wheeze, receipt of antibiotics or hospital admission. Longitudinal studies should further investigate these findings.

Our study has strengths and some limitations. We measured lung function in a large number of extremely prematurely born individuals at 11–14 years of age. The cohort were assessed for exposure to second hand smoke in the family home and a urinary cotinine assessment provided a quantifiable measurement of smoke exposure. Maternal smoking antenatally was assessed by self report and it is possible that some mothers did not respond appropriately, but that would have reduced any impact we note. Furthermore, we did not record how many cigarettes they smoked so cannot comment on whether there was a relationship between the number of cigarettes and LCI results. It has been shown, however, that although mothers do usually say whether they smoke or not, they underestimate how many cigarettes they smoke [34]. We did not find a difference in birthweight in the infants of mothers who smoked versus those that did not reporting smoking, this may be because the children were born extremely preterm. The raw results of the LCI measurements were not stored or accessible, as a consequence further analysis including slope III analysis could not be performed. Active vaping data were not collected and thus was not assessed in our analysis.

In conclusion, extremely prematurely born children exposed to antenatal maternal smoking had a higher lung clearance index indicating greater ventilation inhomogeneity as compared to preterm counterparts unexposed. Furthermore, very prematurely born children exposed to antenatal maternal smoking were more likely to be active smokers at 11–14 years of age and have a household member who smoked both of which may have impaired their lung function.

## Author Contributions

Allan Jenkinson statistical analysis, manuscript writing. Sanja Zivanovic data collection, manuscript review. Christopher Harris data collection, manuscript review. Theodore Dassios statistical analysis, manuscript writing, manuscript review. Anne Greenough conceptualization, manuscript review.

## Ethics Statement

The original UKOS trial was granted ethical approval by the South Thames Multicentre Research Ethics Committee. Follow‐up study at 11–14 years was approved by South West London National Research Ethics Service Committee.

## Conflicts of Interest

The authors declare no conflicts of interest.

## Data Availability

The data that support the findings of this study are available on request from the corresponding author. The data are not publicly available due to privacy or ethical restrictions.
